# Gluten-Free Diet Knowledge and Adherence in Adolescents with Celiac Disease: A Cross-Sectional Study

**DOI:** 10.1097/PG9.0000000000000330

**Published:** 2023-06-26

**Authors:** Katherine Pohoreski, Simonne L Horwitz, Dominica Gidrewicz

**Affiliations:** From the * Department of Pediatrics, University of Calgary, Alberta Children’s Hospital, Calgary, AB, Canada; † Department of Pediatrics, University of Toronto, The Hospital for Sick Children, Toronto, ON, Canada; ‡ Department of Pediatrics, Section of Pediatric Gastroenterology, Hepatology & Nutrition, University of Calgary, Calgary, AB, Canada.

**Keywords:** patient education, pediatrics, dietary management

## Abstract

**Objectives:**

This study examined the relationship between knowledge of, and adherence to, the gluten-free diet (GFD) in a local population of adolescents with celiac disease (CD). The secondary objectives were to identify information sources used to learn about the GFD and to compare adolescents’ and parents’ knowledge of the GFD.

**Methods:**

Adolescents (12–17 years) with CD and their parents from pediatric gastroenterology clinics in Calgary, Alberta, completed an online survey containing a knowledge assessment (Gluten-Free Diet Quiz [GFD-Q]), an adherence scale, questions about GFD information sources, and demographic/clinical information. GFD-Q scores were deemed “sufficient knowledge” with correct identification of 3/3 gluten-containing foods, ≥4/7 gluten-free foods, and ≥ 4/7 foods that may contain gluten; otherwise, scores were termed “insufficient knowledge”.

**Results:**

Of the 40 adolescent-parent pairs, 15 of 40 adolescents (37%) had sufficient knowledge, and 25 of 40 adolescents (63%) had insufficient knowledge. Within the insufficient knowledge group, 14 of 25 (56%) did not correctly identify enough allowed gluten-free foods. Parents scored higher on the GFD-Q (67% had sufficient knowledge). Adolescents reported overall adherence to the GFD (88%), with adherence being similar between the sufficient and insufficient knowledge groups (80% versus 92%). The most helpful information sources included physicians, another person with CD, parent(s), and Google; apps were infrequently used.

**Conclusion:**

Adolescents report good adherence; however, they struggle with GFD knowledge, particularly in identifying gluten-free foods. Further research is required to explore GFD educational tools, including mobile apps and dietician-led teaching sessions.

**What Is Known**

- Lifelong adherence to a strict gluten-free diet (GFD) is the only available treatment for celiac disease (CD).
- Adolescents with CD are at increased risk of GFD nonadherence.
- Knowledge of the GFD correlates with good adherence in adults; however, little is known about this in pediatric patients.

**What Is New**

- Adolescents with insufficient knowledge of the GFD have difficulty identifying allowable gluten-free items but can correctly identify gluten-containing foods to avoid.
- Despite having insufficient knowledge of GFD, adolescents report good dietary adherence.
- Further research is needed to explore educational strategies to enhance adolescent GFD knowledge.

## INTRODUCTION

Celiac disease (CD) is an autoimmune inflammatory enteropathy of the small intestine triggered by the ingestion of gluten, occurring in 1% of the general population (1). Gluten is a protein mainly found in wheat, barley, and rye. Following the diagnosis of CD, patients must follow a lifelong, strict gluten-free diet (GFD) (2); however, adhering to the diet can be challenging.

A recent systematic review of pediatric patients with CD in North America reported GFD adherence rates ranging from 23% to 98%, with a median adherence rate of 79% (3). Adolescents were at the greatest risk of nonadherence, with common issues including availability, cost, and labeling of gluten-free food, with the lowest adherence rates occurring at social events (4). Adolescents who are nonadherent to the GFD experience greater disease burden, poorer quality of life, and more symptoms (5), creating challenges for both patients and healthcare providers in the transition from pediatric to adult care. The World Gastroenterology Organization states that adolescents with CD are “at risk of medical neglect”, given parental oversight and less self-reliance in managing their disease (6), which reinforces the importance of a stable and effective transition period (7,8) and highlights the need for adolescents to gradually assume responsibility in self-managing their CD, facilitated by education about the GFD (9).

Although few studies have evaluated knowledge of GFD in patients with CD, adult studies have shown that adequate dietary knowledge correlates with compliance (10,11). While pediatric literature is limited, studies suggest that a lack of knowledge has been associated with poor compliance (12,13). Several studies reinforce the need for further research in adolescents given low adherence rates, with an emphasis on health education (3,9,14).

The primary aim of this study was to examine the relationship between knowledge of, and adherence to, the GFD in a local population of adolescents with CD. The secondary aims were to identify the information sources used to learn about the GFD and to compare adolescents’ and parents’ knowledge of the GFD.

## METHODS

### Study Design and Participants

A single-center, cross-sectional survey study of 40 adolescents with CD was performed between October 2020 and December 2021 at 2 pediatric gastroenterology clinics in Calgary, Alberta, Canada, that serve all children in central and southern Alberta. The inclusion criteria were as follows: 12–17 years of age, reported diagnosis of CD (serological or histological), and disease management with the GFD for at least 6 months. A parent of each adolescent from the same household also participated in the study. Participant pairs (adolescent and respective parent) were excluded if they did not read or understand English (as the survey was in English), or if they were unable to independently complete the survey (ie, significant developmental delay as determined by the physician).

Recruitment materials included study posters and leaflets posted in pediatric gastroenterology clinics. Eligible patients who were seen in the clinic at a follow-up appointment to review dietary adherence and/or discuss recent serology (either within the first year of diagnosis or on an annual follow-up basis) were provided with consent to contact cards by clinic nurses and physicians. Enrollment was completed by the study personnel over the phone within a few weeks of the clinic appointment, and the participants completed the survey via an emailed online link, with each adolescent and their parent independently submitting their respective surveys. Implied consent and assent were obtained on the first page of the questionnaire. Two reminders were sent 1 week apart, and the survey remained open for a 2-month period.

Upon survey submission, the adolescent surveys and their respective parent surveys were linked using unique anonymized identifiers to capture all required data. Any incomplete data set for an adolescent-parent pair (ie, either the adolescent or parent did not complete their survey) was not used in the final analysis. Data were captured using Qualtrics software (Qualtrics, Provo, UT), version October 2020. The survey was designed by the study team, included both demographic and self-reported clinical information, and was pretested for readability and comprehension by 17 members of the Child and Youth Advisory Council of Calgary. Ethics approval was obtained from the Conjoint Health Research Ethics Board of the University of Calgary (REB20-0315).

### Survey Measures

#### Knowledge

Knowledge of the GFD was assessed using a gluten-free diet Quiz (GFD-Q) based on a previous questionnaire developed by Silvester et al (15), which tests the ability to identify 17 foods as either gluten-free (foods allowed), possibly containing gluten (foods to question), or certainly containing gluten (foods not allowed). After consultation with a CD dietician, the food list was altered and simplified to better suit our study population. For example, we substituted several “foods to question” to highlight common foods consumed by adolescents in the North American diet (potato chips, licorice, French fries, burger [meat], and barbeque sauce). We simplified the food list to include rice (instead of glutinous rice), oats (instead of oatmeal), and malt (instead of malt vinegar). We chose to add maltodextrin as an allowed food given that it was highlighted as a common source of confusion for patients and their families within our clinic. Finally, we chose to highlight wheat, barley, and malt as our 3 “foods not allowed” (as opposed to spelt, egg noodles, and malt vinegar), given that these are common red-flag ingredients in the North American adolescent diet.

The GFD-Q was scored using a rubric designed by the study personnel to divide participants into sufficient versus insufficient knowledge of the GFD. Sufficient knowledge was based on correctly identifying 3 of 3 foods to avoid, at least 4 of 7 foods to question, and at least 4 of 7 foods allowed. Failure to meet any of these criteria was deemed insufficient knowledge. The GFD is strict, with no gluten permitted, which is why individuals must identify all 3 foods to avoid with the other 2 categories allowing for a majority of correct responses. The quiz was based on information available on the Canadian Celiac Association website (16) and was reviewed by content experts (pediatric gastroenterologist, CD dietician, and study team member with CD).

#### Adherence

Adherence to the GFD was measured using the Biagi score, which is a tool that uses 4 simple and reliable questions on GFD compliance (17). The score has been validated in the adult population against known markers of nonadherence (findings on intestinal biopsy or serology), showing that lower adherence scores correspond with the persistence of villous atrophy or positive endomysial antibodies. The score can be used regardless of ethnic group, as it focuses on the strategy used to avoid gluten. The responses translate into a final score out of 4: 0–1 (do not follow a strict GFD), 2 (follow a GFD but with important errors that need correction), and 3–4 (follow a strict GFD). Although this tool is not yet validated for use in pediatrics, its questions are asked on simplified dietary review in our clinic and are useful in assessing compliance with the GFD in patients who have been instructed on the GFD and how to navigate it. Expert dietary interview, the gold-standard method for addressing adherence (18), is time-consuming, and self-reported adherence is not always reliable. Of note, given that adherence has the potential to fluctuate, we specified that adolescents answer the Biagi survey questions while reflecting on the preceding 2-week period. We did not complete a separate dietary review in addition to the Biagi survey questions.

#### Information Sources

Both adolescent and parent participants classified a list of GFD information sources as “helpful”, “not helpful”, or “not used” based on their prior experiences. The list included a primary physician (family physician or pediatrician), gastroenterologist, dietician, alternative health care provider, celiac association, another individual with CD, parent(s), special event, Google (online search engine), apps (downloadable custom software), and books.

### Study Analysis

The participant pairs were assigned a unique, anonymous identification number. The data were exported to Microsoft Excel for Microsoft 365 MSO (Version 2209 Build 16.0.15629.20152) for analysis by 2 independent reviewers. Data were analyzed using descriptive statistics and categorized based on sufficient versus insufficient knowledge, with the primary outcome of assessing the proportion of adolescents who reported adherence to the GFD.

We received statistical advice through the Resident Research Course of the Cumming School of Medicine at the University of Calgary. We assumed 80% adherence in the sufficient knowledge group and 25% adherence in the insufficient knowledge group (alpha level 0.05, power of 80%). A total sample size of 40 (32 in the sufficient knowledge group and 8 in the insufficient knowledge group) would allow us to find such a difference if it is as big as stated.

## RESULTS

Enrollment included 57 adolescent-parent pairs. After data were reviewed, 17 adolescent-parent pairs were excluded from the analysis due to incomplete surveys. The final data analysis was completed for 40 adolescent-parent pairs.

### Demographics

The study included 40 adolescents with CD and their parents. The adolescents had a mean age of 14 years, ranging from 12 to 17 years. There were 30 females and 10 males, with the majority being Caucasian (37/40). Most adolescents had been following the GFD for over 2 years (26/40) and endorsed symptoms before the diagnosis of CD (34/40). The parent respondents were primarily mothers (35/40). Overall, the 2 groups (sufficient knowledge and insufficient knowledge) were similar; however, there was a higher proportion of females than males in the sufficient knowledge group and more diverse ethnic representation in the insufficient knowledge group (Table 1).

**TABLE 1.** Demographics and clinical characteristics of 40 adolescents with celiac disease with sufficient vs insufficient knowledge based on Gluten-Free Diet Quiz results

	Total adolescents (*n* = 40)	Sufficient knowledge (*n* = 15)	Insufficient knowledge (*n* = 25)
**Demographics**			
Adolescent age (12–17)			
Median	14	14	14
Mean	14	15	14
Gender			
Female	30 (75%)	13 (87%)	17 (68%)
Male	10 (25%)	2 (13%)	8 (32%)
Grade			
Grade 6 or less	2 (5%)	0	2 (8%)
Grade 7 to 9	18 (45%)	8 (53%)	10 (40%)
Grade 10 to 12	20 (50%)	7 (47%)	13 (52%)
Survey respondent			
Mother	35 (87%)	15 (100%)	20 (80%)
Father	4 (10%)	0	4 (16%)
Other	1 (3%)	0	1 (4%)
Living area			
Urban	14 (35%)	7 (47%)	7 (28%)
Suburban	20 (50%)	5 (33%)	15 (60%)
Rural	6 (15%)	3 (20%)	3 (12%)
Ethnicity (select all that apply)			
White/Caucasian	37 (93%)	15 (100%)	22 (88%)
North American	24 (60%)	11 (73%)	13 (52%)
European	11 (28%)	3 (20%)	8 (32%)
Latin American	4 (10%)	1 (7%)	3 (12%)
Asian (East Asian)	1 (3%)	0	1 (4%)
Indigenous/Aboriginal	1 (3%)	0	1 (4%)
**Clinical characteristics**			
Time on the GFD			
6–12 months	6 (15%)	1 (7%)	5 (20%)
1–2 years	8 (20%)	3 (20%)	5 (20%)
Over 2 years	26 (65%)	11 (73%)	15 (60%)
Symptoms pre–CD diagnosis			
Yes	34 (85%)	14 (93%)	20 (80%)
No	5 (13%)	1 (7%)	4 (16%)
Unsure	1 (3%)	0	1 (4%)
Method of CD diagnosis			
Blood test only	18 (45%)	6 (40%)	12 (48%)
Blood test + biopsy	13 (33%)	6 (40%)	7 (28%)
Blood test + biopsy + genetic test	3 (8%)	1 (7%)	2 (8%)
Blood test + genetic test	3 (8%)	1 (7%)	2 (8%)
Biopsy only	3 (8%)	1 (7%)	2 (8%)

CD = celiac disease; GFD = Gluten-Free Diet.

### Knowledge

Among the adolescents, 26 of 40 (65%) correctly identified at least 4 of 7 gluten-free foods, 32 of 40 (80%) correctly identified at least 4 of 7 foods that may contain gluten, and 31 of 40 (78%) correctly identified all 3 gluten-containing foods. Therefore, based on the overall scoring rubric of the GFD-Q, 15 of 40 (37%) adolescents had sufficient knowledge, and 25 of 40 (63%) had insufficient knowledge. Adolescent scores ranged from 8 to 16 out of 17 on the GFD-Q, with a mean score of 11.4 and a median score of 11.

**FIGURE 1.**
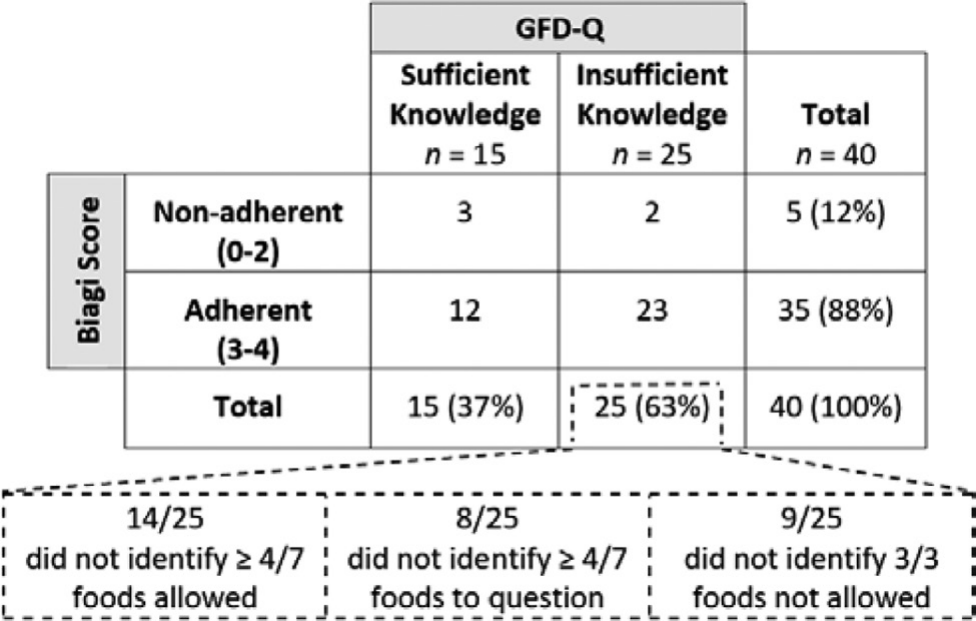
Comparison of knowledge and adherence in a local population of 40 adolescents with celiac disease. Knowledge was assessed with the Gluten-Free Diet Quiz (GFD-Q) and adherence was based on the Biagi score. Subcategory analysis is represented within the dashed lines. Percentages were rounded to sum to 100.

Of the 25 adolescents with insufficient knowledge, 14 of 25 (56%) did not correctly identify at least 4 of 7 gluten-free foods, 8 of 25 (32%) did not correctly identify at least 4 of 7 foods that may contain gluten, and 9 of 25 (36%) did not correctly identify all 3 gluten-containing foods (Fig. 1). In analyzing all 40 adolescent responses, of the 7 gluten-free foods included in the GFD-Q, maltodextrin, balsamic vinegar, and buckwheat were correctly identified by 7 of 40 (18%), 12 of 40 (30%), and 13 of 40 (33%) adolescents, respectively. Corn tortilla and cocoa were misidentified by 13 of 40 (33%) and 16 of 40 (40%) adolescents, respectively. Rice was misidentified by 10 of 40 (25%) adolescents. Milk was the only gluten-free item correctly identified by 100% of the adolescents. Of the foods that may contain gluten, licorice, and soy were instead classified as gluten-containing foods (75% and 30% of the time, respectively); burger (meat) was thought to be gluten-free by 43% of adolescents. Most adolescents correctly identified the gluten-containing foods: wheat (98%), barley (98%), and malt (80%) (Table 2).

**TABLE 2.** Detailed responses to the Gluten-Free Diet Quiz by 40 adolescents with celiac disease. Participants were asked to identify each food as a food allowed, food to question, or food to avoid. Bolded values indicate correct responses.

	Foods allowed	Foods to question	Foods not allowed
Gluten-free foods (Allowed)			
Milk	**100%**	0%	0%
Buckwheat	**33%**	15%	53%
Rice	**75%**	23%	3%
Maltodextrin	**18%**	55%	28%
Balsamic vinegar	30%	58%	13%
Cocoa powder	**60%**	40%	0%
Corn tortilla	**68%**	33%	0%
May contain gluten (Question)			
Potato chips	18%	**83%**	0%
Soy sauce	0%	**70%**	30%
Oats	5%	**75%**	20%
Licorice	0%	**25%**	75%
Barbeque sauce	10%	**85%**	5%
French fries	15%	**83%**	3%
Burger (meat)	43%	**58%**	0%
Contain gluten (Not Allowed)			
Malt	0%	20%	**80%**
Wheat	3%	0%	**98%**
Barley	0%	3%	**98%**

Percentages may not sum to 100 due to rounding.

Parents scored higher than adolescents on the GFD-Q, with 27 of 40 (67%) having sufficient knowledge and higher scores across all subcategories. Parent scores on the GFD-Q ranged from 8 to 15 of 17, with a mean score of 12.9 and a median score of 13. Interestingly, of the 37% of adolescents with sufficient knowledge, most parents also had sufficient knowledge (87%). Of the adolescents with insufficient knowledge, only 56% of their parents had sufficient knowledge (See, Supplemental Digital Content Table 1, http://links.lww.com/PG9/A115 and Supplemental Digital Content 2 Figure, http://links.lww.com/PG9/A116).

### Adherence

Using the Biagi score, 35 of 40 (88%) adolescents reported adherence to the GFD with a score of 3 or 4. The remaining 5 adolescents, all female, were classified as nonadherent. One adolescent reported a score of 1, indicating voluntary gluten consumption.

### Comparison of Knowledge and Adherence

Of the 15 adolescents with sufficient knowledge of the GFD on the GFD-Q, 12 of 15 (80%) reported adherence to the GFD. Of the 25 adolescents with insufficient knowledge, 23 of 25 (92%) reported adherence to the GFD. Regardless of the GFD-Q knowledge results, the reported adherence rates were similar between the groups (Fig. 1).

### Information Sources

**FIGURE 2.**
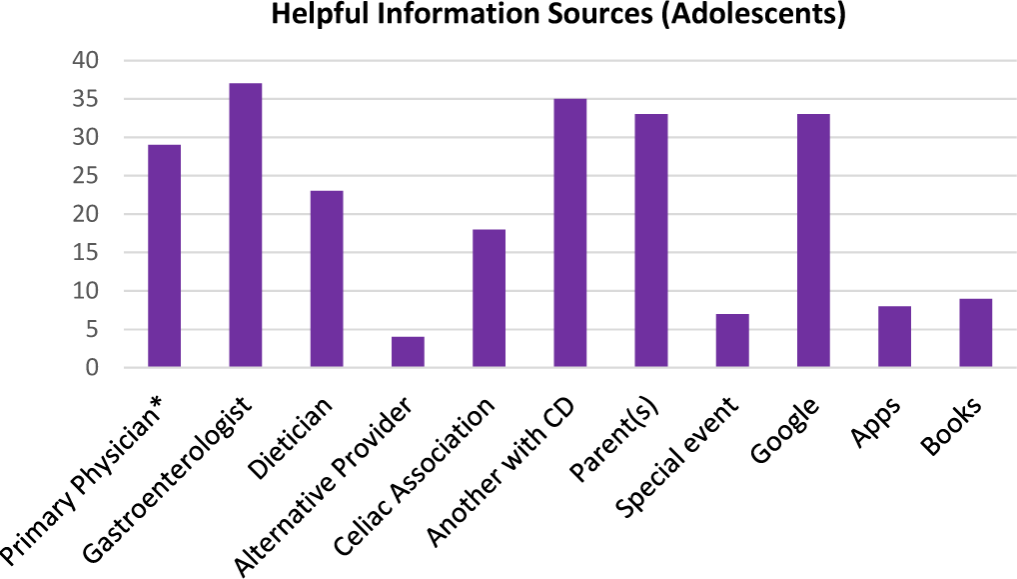
Helpful information sources identified by 40 adolescents with celiac disease (CD) to learn about the gluten-free diet. *Family Physician or Pediatrician.

The most frequently reported helpful resources by adolescents included a gastroenterologist (37/40), another person with CD (35/40), parent(s) (33/40), Google (33/40), and a primary care physician (29/40). Books, special events, and alternative providers were not frequently considered helpful. Notably, apps were helpful in only 8 of 40 (20%) adolescents, and the remaining adolescents indicated that apps were generally not used (Fig. 2).

Similar trends for helpful information sources were reported by parents (See Supplemental Digital Content 3 Figure, http://links.lww.com/PG9/A117).

## DISCUSSION

Based on the available literature, this study is the first North American study to assess knowledge of, and adherence to, the GFD in adolescents. Adolescents found to have insufficient knowledge of the GFD had surprisingly high rates of self-reported GFD adherence (92%), similar to adolescents with sufficient knowledge (80%). This differs from previous adult studies that showed positive correlations between GFD knowledge and adherence (13). However, the participants in our study with insufficient knowledge generally failed the GFD-Q only because they were more cautious in their responses; despite correctly identifying gluten-containing foods to avoid, they struggled to identify allowable gluten-free foods. This helps explain the overall high rates of adherence while also demonstrating that adolescents need education on gluten-free items so that they can feel confident in choosing safe foods to eat.

All of the gluten-containing foods in our survey (wheat, barley, and malt) were correctly identified by 78% of adolescents, similar to the results of a study of Canadian adults with biopsy-confirmed CD and/or dermatitis herpetiformis, in which 81% of respondents correctly identified 6 of 7 gluten-containing foods (19). The ability of adolescents to accurately identify red-flag foods is reassuring, as maintaining a strict GFD is currently the only available treatment for CD. Despite this, only 37% (15/40) of adolescents passed the GFD-Q, as they generally struggled to correctly identify the gluten-free items. Maltodextrin, balsamic vinegar, and buckwheat were the most commonly misidentified, with less than a third of adolescents correctly identifying these items as safe foods, highlighting the need for further education on ingredients that may be unfamiliar to adolescents. Although rice was less commonly misidentified, as a gluten-free carbohydrate staple, it is worrisome that 25% of adolescents were unable to identify it as a safe food.

Although we classified corn tortilla as an allowable gluten-free item, the Canadian Celiac Association considers it a “food to question” (16). If we had accepted this as a correct response, another three adolescents would have been categorized with sufficient knowledge based on the accuracy of their other GFD-Q responses. Additionally, most adolescents misidentified licorice as “foods not allowed”; we classified licorice as “foods to question” as, although it typically uses wheat as a binding ingredient, there are gluten-free options available (20,21). If we had also accepted licorice as an unallowable food, then another two adolescents would have passed the GFD-Q. Accepting these alternate answers for licorice and corn tortilla, thereby considering 5 more adolescents to have passed the GFD-Q, would have resulted in a 13% increase in the number of adolescents with sufficient knowledge, or a 50% GFD-Q pass rate (20/40). Nevertheless, even if there was a higher pass rate, the overall trend of adolescents struggling to identify gluten-free foods remains clear.

In our study, 88% of adolescents reported GFD adherence, as measured using the Biagi score. The importance of adhering to the GFD cannot be overstated. In addition to gastrointestinal and extraintestinal symptoms, patients with uncontrolled CD are at increased risk of developing nutritional deficiencies due to inflammation leading to villous atrophy (2). While a GFD can restore damaged villi (22), a diet that is too restrictive may also lead to nutritional deficiencies due to inadequate intake (15). Compounding this is the fact that gluten-free products often have lower levels of various vitamins and minerals, including folate, thiamin, and other B vitamins, compared to their gluten-containing equivalents (15,23). Education on CD and the GFD has traditionally focused on foods to avoid, but the GFD-Q results reveal a concerning trend of relatively poor knowledge about gluten-free options available to diversify the diet. This study highlights that adolescents require further education on allowable foods to avoid unnecessarily restricting their diet and nutritional intake.

Adolescents identified other people, including their pediatric gastroenterologist, primary care physician (family physician or pediatrician), and parent(s), as helpful information sources. Given that adolescent knowledge appears to be positively correlated with parental knowledge, this demonstrates the important role that parents play in adolescent education. Parents tended to score better overall than adolescents in terms of knowledge (See, Supplemental Digital Content 1 Table, http://links.lww.com/PG9/A115); this difference could be due to greater parent attendance at the gluten-free family teaching sessions provided by our clinic dieticians. We do not have modules specific to adolescent education at these classes, so adolescents often do not receive additional dedicated teaching beyond what is discussed at their clinic appointments.

Interestingly, although dieticians lead the GFD teaching classes at the study clinics, dieticians were only classified as helpful by 57% of the adolescents surveyed. A recent North American study found that in-person small-group GFD teaching sessions led by dieticians successfully increased GFD and CD knowledge in children over 8 years old based on parent reports (24), highlighting an opportunity for ongoing research and improvement at the study clinics. Aside from Google, adolescents did not report using online resources such as apps (including social media outlets) as a GFD information source, which is surprising given their overall reliance on the Internet. An estimated 90% of adolescents use social media apps, with 51% of those surveyed reporting visiting a social media website at least daily (25), and although data remains limited, apps have been evaluated within adolescents with chronic diseases (eg, type 1 diabetes, asthma, beta thalassemia major and sickle cell disease, and epilepsy), identifying an opportunity for adolescent-oriented educational resources (26).

This study is potentially limited by recruitment bias, as those who are knowledgeable about and/or adherent to the GFD may have been more inclined to participate. Another potential limitation is the inability of participants to clarify survey questions given their online nature, which could potentially affect their responses. While our goal was to collect survey information as close to the clinic follow-up as possible, a further limitation is that the timing of participant response was variable, and we did not collect information related to active CD symptomatology at the time of survey submission. Additionally, the foods listed in the GFD-Q were chosen by content experts but may not be representative of foods that adolescents eat, possibly influencing their knowledge scores. Finally, due to the small sample size, we did not analyze whether demographic factors or resources used affected GFD-Q pass rates, although there did not appear to be any obvious trends. The small sample size may also limit the generalizability of this study, especially to non-English speaking populations.

In conclusion, the results of this study show that although adolescents typically adhere to the GFD by avoiding gluten-containing foods, they struggle to confidently identify allowable gluten-free foods. This may lead to excessive limitation of the foods they consume, which may increase their risk of developing nutritional deficiencies and create challenges for food choices, especially in social settings. Further education on the nuances of the GFD, particularly with respect to safe, gluten-free foods, is therefore required, especially as adolescents take increasing ownership of their dietary choices. Opportunities for further research include the exploration of mobile apps as GFD educational tools and the assessment of adolescent participation and knowledge acquisition in dietician-led GFD teaching sessions, with the goal of supporting adolescents in independently managing their CD as they transition into adulthood.

## ACKNOWLEDGMENTS

We thank the Canadian Celiac Association, recently named Celiac Canada, for funding this study as well as the Silvester et al. group for their previous work, which was a foundation for the concept of this study and the basis of our knowledge quiz.

## Supplementary Material

## References

[1] Guandalini S Assiri A. Celiac disease: a review. JAMA Pediatr. 2014;168:272–278. 24395055 10.1001/jamapediatrics.2013.3858

[2] Norström F Sandström O Lindholm L. A gluten-free diet effectively reduces symptoms and health care consumption in a Swedish celiac disease population. BMC Gastroenterol. 2012;12:125. 22984893 10.1186/1471-230X-12-125 PMC3482575

[3] Myléus A Reilly NR Green PH. Rate, risk factors and outcomes of non-adherence in pediatric patients with celiac disease: a systematic review. Clin Gastroenterol Hepatol. 2020;18:562–573. 31173891 10.1016/j.cgh.2019.05.046

[4] MacCulloch K Rashid M. Factors affecting adherence to a gluten-free diet in children with celiac disease. Paediatr Child Health. 2014;19:305–309. 25332660 10.1093/pch/19.6.305 PMC4173957

[5] Wagner G Berger G Sinnreich U. Quality of life in adolescents with treated coeliac disease: influence of compliance and age at diagnosis. J Pediatr Gastroenterol Nutr. 2008;47:555–561. 18955861 10.1097/MPG.0b013e31817fcb56

[6] Bai JC Ciacci C. World gastroenterology organisation global guidelines: celiac disease February 2017. J Clin Gastroenterol. 2017;51:755–768. 28877080 10.1097/MCG.0000000000000919

[7] O’Leary C Wieneke P Healy M. Celiac disease and the transition from childhood to adulthood: a 28-year follow-up. Am J Gastroenterol. 2004;99:2437–2441. 15571593 10.1111/j.1572-0241.2004.40182.x

[8] Mozer-Glassberg Y Zevit N Rosenbach Y. Follow-up of children with celiac disease - lost in translation? Digestion. 2011;83:283–287. 21282953 10.1159/000320714

[9] Ludvigsson JF Agreus L Ciacci C. Transition from childhood to adulthood in coeliac disease: the Prague consensus report. Gut. 2016;65:1242–1251. 27196596 10.1136/gutjnl-2016-311574 PMC4975833

[10] Roma E Roubani A Kolia E. Dietary compliance and life style of children with coeliac disease. J Hum Nutr Diet. 2010;23:176–182. 20163513 10.1111/j.1365-277X.2009.01036.x

[11] Zingone F Massa S Malamisura B. Coeliac disease: factors affecting the transition and a practical tool for the transition to adult healthcare. United European Gastroenterol J. 2018;6:1356–1362. 10.1177/2050640618787651 PMC6206529 30386608

[12] Mayer M Greco L Troncone R. Compliance of adolescents with coeliac disease with a gluten free diet. Gut. 1991;32:881–885. 1885070 10.1136/gut.32.8.881 PMC1378956

[13] Ljungman G Myrdal U. Compliance in teenagers with coeliac disease—a Swedish follow-up study. Acta Paediatr. 1993;82:235–238. 8495075 10.1111/j.1651-2227.1993.tb12649.x PMC7188325

[14] Martínez-Martinez MI Alegre-Martínez A García-Ibánez J. Quality of life in people with coeliac disease: psychological and Socio-economic aspects. Endocr Metab Immune Disord Drug Targets. 2019;19:116–120. 30033882 10.2174/1871530318666180723100003

[15] Silvester JA Weiten D Graff LA. Is it gluten-free? Relationship between self-reported gluten-free diet adherence and knowledge of gluten content of foods. Nutrition. 2016;32:777–783. 27131408 10.1016/j.nut.2016.01.021 PMC5457910

[16] Canadian Celiac Association and Dieticians of Canada. Gluten-Free Eating [Internet]. Published May 15, 2018. Available at: https://www.celiac.ca/wp-content/uploads/2018/05/Gluten-Free-Eating-PEN-Document.pdf. Accessed October 15, 2022.

[17] Biagi F Bianchi PI Marchese A. A score that verifies adherence to a gluten-free diet: a cross-sectional, multicentre validation in real clinical life. Br J Nutr. 2012;108:1884–1888. 22321199 10.1017/S0007114511007367

[18] Pietzak MM. Follow-up of patients with celiac disease: achieving compliance with treatment. Gastroenterology. 2005;128(4 Suppl 1):S135–S141. 15825121 10.1053/j.gastro.2005.02.025

[19] Zarkadas M Dubois S MacIsaac K. Living with coeliac disease and a gluten-free diet: a Canadian perspective. J Hum Nutr Diet. 2013;26:10–23. 23157646 10.1111/j.1365-277X.2012.01288.x

[20] Made How. How Products are Made: Licorice [Internet]. Available at: http://www.madehow.com/Volume-4/Licorice.html. Accessed October 15, 2022.

[21] Canadian Celiac Association. Gluten-Free Product Finder [Internet]. Available at: https://www.celiac.ca/living -gluten-free/gf-product-finder/. Accessed October 15, 2022.

[22] Verma AK. Nutritional deficiencies in celiac disease: current perspectives. Nutrients. 2021;13:4476. 34960029 10.3390/nu13124476 PMC8703793

[23] Theethira TG Dennis M. Celiac disease and the gluten-free diet: consequences and recommendations for improvement. Dig Dis. 2015;33:175–182. 25925920 10.1159/000369504

[24] Matschull L Martin N Goday P. Evaluation of in-person, gluten-free diet education for children with celiac disease. JPGN Rep. 2022;3:e218. 37168641 10.1097/PG9.0000000000000218 PMC10158309

[25] American Academy of Child and Adolescent Psychiatry. Facts for Families: Social Media and Teens [Internet]. Published March 2018. Available at: https://www.aacap.org/AACAP/Families_and_Youth/Facts_for_Families/FFF-Guide/Social-Media-and-Teens-100.aspx. Accessed October 15, 2022.

[26] Virella Pérez YI Medlow S Ho J. Mobile and web-based apps that support self-management and transition in young people with chronic illness: systematic review. J Med Internet Res. 2019;21:e13579. 31746773 10.2196/13579 PMC6893564

