# Supplementary material for: Gluten-Free Diet Knowledge and Adherence in Adolescents with Celiac Disease: A Cross-Sectional Study
Source: JPGN Rep. 2023 Jun 26;4(3):e330. doi: 10.1097/PG9.0000000000000330 (PMC10435025; doi:10.1097/PG9.0000000000000330)
Supplement: Supplementary file 1 [file pg9-4-e330-s001.pdf]

Table, Supplemental Digital Content 1: Detailed responses to the Gluten-Free Diet Quiz by 40 parents of adolescents with celiac disease. Participants were asked to identify each food as a food allowed, food to question, or food to avoid. Bolded values indicate correct responses.

	Foods Allowed	Foods to Question	Foods Not Allowed
Gluten-free foods (Allowed)			
Milk	**98%**	0%	3%
Buckwheat	**48%**	13%	40%
Rice	**73%**	28%	0%
Maltodextrin	**50%**	30%	20%
Balsamic vinegar	**53%**	35%	13%
Cocoa powder	**58%**	43%	0%
Corn tortilla	**53%**	48%	0%
May contain gluten (Question)			
Potato Chips	3%	**98%**	0%
Soy sauce	0%	**73%**	28%
Oats	3%	**90%**	8%
Licorice	3%	**35%**	63%
Barbeque sauce	0%	**93%**	8%
French Fries	0%	**98%**	3%
Burger (meat)	15%	**85%**	0%
Contain gluten (Not Allowed)			
Malt	0%	5%	**95%**
Wheat	0%	0%	**100%**
Barley	0%	3%	**98%**

Percentages may not sum to 100 due to rounding.
