# Supplementary material for: Gluten-Free Diet Knowledge and Adherence in Adolescents with Celiac Disease: A Cross-Sectional Study
Source: JPGN Rep. 2023 Jun 26;4(3):e330. doi: 10.1097/PG9.0000000000000330 (PMC10435025; doi:10.1097/PG9.0000000000000330)
Supplement: Supplementary file 2 [file pg9-4-e330-s002.pdf]

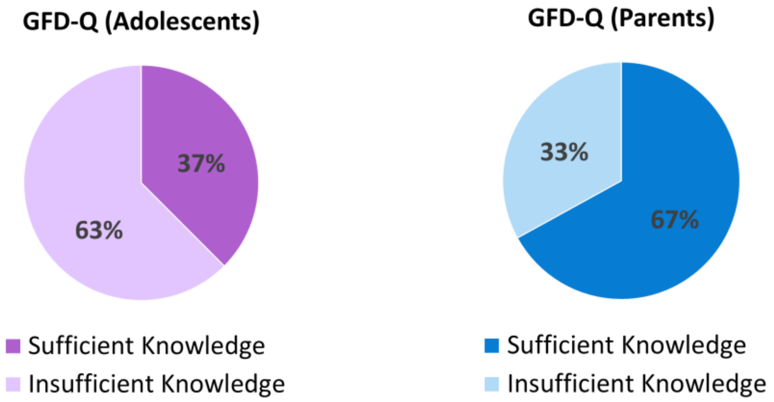

Figure, Supplemental Digital Content 2: Comparison of adolescent and parent Gluten Free Diet Quiz (GFD-Q) results, with proportions of sufficient vs. insufficient knowledge identified for each group. GFD-Q scores were categorized as "sufficient knowledge" based on correctly identifying 3/3 gluten-containing foods, ≥ 4/7 gluten-free foods, and ≥ 4/7 foods that may contain gluten, otherwise were termed "insufficient knowledge". Parents scored higher (67% with sufficient knowledge) than adolescents (37% with sufficient knowledge).
