# Supplementary material for: Gluten-Free Diet Knowledge and Adherence in Adolescents with Celiac Disease: A Cross-Sectional Study
Source: JPGN Rep. 2023 Jun 26;4(3):e330. doi: 10.1097/PG9.0000000000000330 (PMC10435025; doi:10.1097/PG9.0000000000000330)
Supplement: Supplementary file 3 [file pg9-4-e330-s003.pdf]

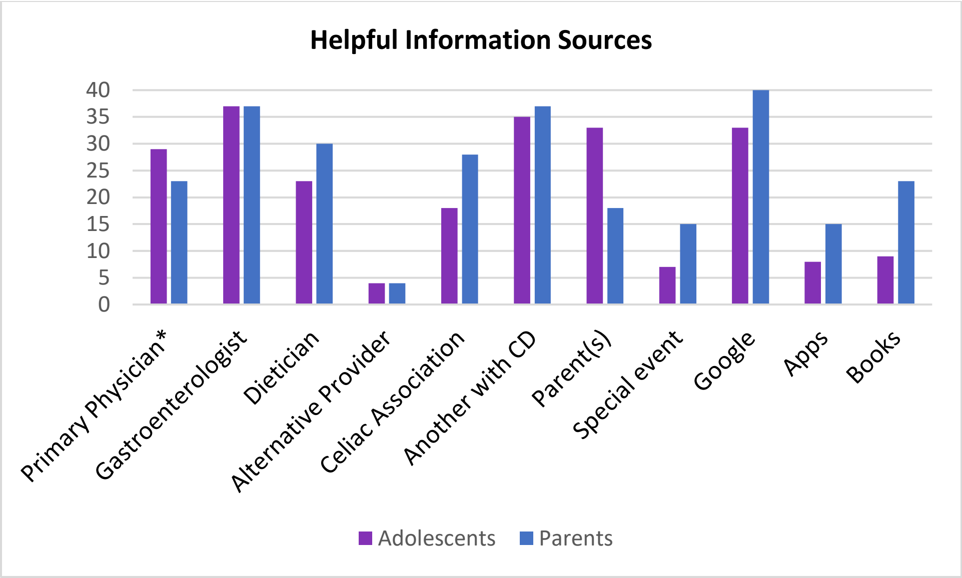

**Family Physician or Pediatrician*

Figure, Supplemental Digital Content 3: Helpful information sources to learn about the gluten-free diet identified by 40 adolescents with celiac disease (CD) and their parents.
